# Leptospirosis presenting as haemolytic uraemic syndrome: a case report

**DOI:** 10.1186/s12882-018-0817-5

**Published:** 2018-01-29

**Authors:** Vasantha Muthu Muthuppalaniappan, Ravindra Rajakariar, Mark John Blunden

**Affiliations:** 1 Department of Renal Medicine and Transplantation, The Royal London Hospital, Barts Health NHS Trust, London, UK; 2 William Harvey Research Instititute, Queen Mary University of London, London, UK

**Keywords:** Acute kidney injury, Acute liver injury, Haemolytic uraemic syndrome, Leptospirosis, Plasma exchange, Case report

## Abstract

**Background:**

Leptospirosis is a rare infectious disease especially in Western Countries. Renal involvement is a recognised complication of leptospirosis but leptospirosis-associated haemolytic uraemic syndrome is extremely rare and to our knowledge has only been reported once, in 1985.

**Case presentation:**

A 29-year-old male was transferred to our Renal Unit with fevers, myalgia and diarrhoeal illness. Laboratory investigations revealed an acute kidney injury, acute liver injury, significantly raised lactate dehydrogenase with marked anaemia, thrombocytopenia and schistocytes on a blood film. A diagnosis of haemolytic uraemic syndrome was made. Surprisingly, the stool culture was negative which led to a suspicion of leptospirosis as one of the differential diagnoses. This was subsequently confirmed by enzyme-linked immunosorbent assay and microscopic agglutination test. He received plasma exchange and antibiotics and made a complete recovery on discharge.

**Conclusion:**

Leptospirosis presenting as haemolytic uraemic syndrome is rare but should be considered in the differential diagnosis especially in the presence of significant liver injury, as current evidence suggests that the disease is re-emerging.

## Background

Leptospirosis is a zoonotic disease caused by spirochetes of the genus *Leptospira* that was first described in 1886 by Weil. It is common in tropical and subtropical environments but a rare finding in the United Kingdom (UK). According to the Health Protection Agency, there are usually less than 40 cases reported per year in England and Wales in humans. However, in 2014, there were 76 confirmed cases compared to 47 in 2013, exceeding the peak reporting of 74 in 2007.

There are over 250 serotypes but the commonest species in UK are *Leptospira hardjo* and *Leptospira icterohaemorrhagiae* [1]. The disease is spread through contact with water or soil contaminated by *leptospira*-infected animals that shed urine into the ecosystem. The contaminated water or soil infects humans via the skin or gastrointestinal route. Current recognised risk factors include occupational exposures, recreational activity, flooding and household environments in close contact with animals.

Leptospirosis is characterised by a wide spectrum of clinical features that range from a subclinical infection and self-limiting illness to multi-organ failure and death. Typical manifestations of leptospirosis fall into four categories: (i) mild influenza-like illness; (ii) Weil’s syndrome characterized by jaundice, renal failure, haemorrhage and myocarditis with arrhythmias; (iii) meningitis / meningoencephalitis; and (iv) pulmonary haemorrhage with respiratory failure [2]. Up to 10% of leptospirosis infections induce multi-organ failure with mortality greater than 50% in Weil’s disease [3].

In this report, we describe an extremely rare presentation of leptospirosis with associated haemolytic uraemic syndrome that to our knowledge has only been reported once, in 1985 by Hanvanich et al.

## Case presentation

A 29-year-old Caucasian male who was previously fit and well was transferred to our renal unit with significant acute kidney injury (AKI).

He first presented to his local hospital with palpitations, a weeklong history of fever, myalgia, progressive generalised muscle weakness and a diarrhoeal illness. He denied any headaches or confusion. His symptoms started approximately 24 h after attending a barbeque. No one else that attended the barbeque was unwell. There was no history of foreign travel or any illicit drug abuse.

He works as an office fitter. He likes fishing regularly but has not indulged in this activity of recent. He admitted to caring for his mother’s pet, a cat, in the past 2 weeks.

On examination, he was clinically jaundiced. His temperature was 38.4**°**c; blood pressure (BP) 121/57 mmHg; and pulse 70 bpm. Cardiovascular, respiratory, abdominal and neurological examination was otherwise unremarkable.

Urinalysis showed presence of blood 3+ and protein 2+. Electrocardiogram only showed sinus tachycardia with no evidence to suggest pericarditis.

Initial investigations on Day 1 of admission revealed a haemoglobin level of 9.4 g/dl, white cell count 11.5 × 10^9^/L and marked thrombocytopenia with a platelet count of 17 × 10^9^/L. His urea was raised at 23.7 mmol/L with a creatinine level of 534 μmol/L. His potassium was 4.1 mmol/L with a bicarbonate level of 18 mmol/L. His liver function test was also deranged with an albumin of 23 g/L, bilirubin 281 μmol/L, alkaline phosphatase (ALP) 58 unit/L and alanine aminotransferase (ALT) of 212 unit/L. International normalized ratio was minimally raised at 1.2 and with a normal prothrombin time of 12.4 s. His creatine kinase was elevated at 8776 unit/L with a normal troponin level. C- reactive protein was also elevated at 200 mg/dL. Both haptoglobin and fibrinogen levels were raised at 466 mg/dL and 5.64 g/L; consistent with an infective process. The lactate dehydrogenase level was significantly raised at 1109 unit/L. C3 level was normal but C4 level was reduced at 0.06 g/L. Autoimmune markers including antinuclear antibody, anti-double-stranded DNA antibodies and antiphospholipid screen were negative.

The initial blood film at the local hospital and a repeat at our tertiary unit showed presence of schistocytes. His direct antiglobulin test screen was negative.

In light of his history of diarrhoea, acute kidney injury, and thrombocytopenia with evidence of haemolysis, a presumptive diagnosis of shiga toxin-producing *Escherichia coli* induced haemolytic uraemic syndrome (HUS) was made. He was given platelet transfusion and initiated on daily plasma exchange (PEX). The decision was also made to prescribe Piperacillin/Tazobactam in view of his raised inflammatory markers, pyrexia and chest x-ray suggesting a hospital-acquired pneumonia. After 12 h of antibiotic administration and PEX on Day 1, he became unwell with evidence of shock. He had further temperature spikes of 38**°c**, BP dropped to 60/40 mmHg and tachycardia of 150 bpm. Repeat investigations showed worsening inflammatory markers and evidence of ongoing haemolysis with marked anaemia. He required inotropic support for 4 h as he was resuscitated with fluid and blood products. He remained profoundly polyuric since admission.

Ultrasound abdomen showed normal sized kidneys and no abnormalities seen in other visceral organs. Echocardiogram was unremarkable.

On Day 3 when both blood and stool cultures were found to be negative, a probability of systemic infection was explored. Serum antibodies for brucella, bartonella, cytomegalovirus, Ebstein-Barr virus, human immunodeficiency virus and hepatitis were negative. A positive Microscopic Agglutination Test (MAT) with raised titres at 1:320 and the presence of anti-*leptospira* IgM by ELISA at a titre of 1:2560 confirmed the suspicion of leptospirosis on illness day 14 (admission day 7).

He continued to improve clinically. A renal biopsy was not attempted due to his presentation with profound thrombocytopenia and his renal function improved rapidly with treatment. He completed 5 days of intravenous antibiotics and 10 days of oral doxycycline. He only received 4 sessions of PEX and never required dialysis. He was discharged on Day 10 with a creatinine level of 82 μmol/L and a platelet count of 294 × 10^9/L. His liver function tests were gradually improving with a bilirubin of 264 μmol/L (peak bilirubin 705 μmol/L), ALT 148 unit/L (peak ALT 212 unit/L) and ALP 147 unit/L (peak ALP 200 unit/L).

## Discussion

Renal involvement in leptospirosis is frequent and ranges from subtle changes with urinary sediment to acute kidney injury (AKI). AKI due to leptospirosis is common in tropical countries but remains a rare cause of AKI in western countries. Most of the published literatures are from Asia. Up to 24% of cases of AKI in South East Asia is caused by leptospirosis [4]. Approximately 10–85% of infected patients develop AKI, 30% of the AKI cases require dialysis and the mortality rate varies between different series at 4–20% [5]. A retrospective analysis of serologically confirmed AKI due to leptospirosis revealed that 63.8% of patients completely recovered their renal function after 90 days but 10.3% were left with persisting mild renal insufficiency [6]. Presence of oligouria is an independent risk factor for mortality [2].

Leptospires can cause renal injury by direct action of leptospires on renal interstitium and tubules, indirect toxicity by activating toll like receptors and protein expression [2, 3, 7]. The main lesion seen in *leptospira* infection is tubulointerstitial nephritis with sparse lymphocytic infiltrates, tubular necrosis and interstitial oedema [2, 7]. Renal tubular dysfunction causing polyuria and hypokalaemia as a result of *leptospira* endotoxin impairing proximal sodium absorption is also often seen in these patients [2, 3, 6, 7].

Thrombocytopenia has been reported to occur in 50% -80% of leptospirosis cases [8]. This can be the result of haemolytic uraemic syndrome (HUS) or thrombotic thrombocytopenic purpura (TTP), which is rare, or in association with AKI as a result of severe endotoxin injury of leptospirosis. Thrombocytopenia is found to be a significant predictor of AKI [3, 7]. The outer membrane of leptospires consists of lipopolysaccharide and outer membrane protein that stimulate the adherence of neutrophils to endothelium and platelet aggregation, suggesting a role in the development of thrombocytopenia [9, 10]

Systemic infections may mimic the presenting clinical features of TTP and leptospirosis associated TTP is well documented in the literature [4, 11]. The Oklahoma TTP-HUS Registry from 1989 to 2010 showed that 7% of patients presenting with clinical features consistent with TTP were subsequently attributed to systemic infection [11]. Initial PEX treatment is appropriate in critically ill patients with diagnostic features of TTP, even if a systemic infection is suspected [11]. At the severe end of the disease spectrum, leptospirosis can present and mimic thrombotic microangiopathies [1]. HUS has been reported in *Leptospira bataviae* infection [12].

The gold standard for diagnosis is a MAT to detect antibody to *leptospira*. This diagnostic method is preferred due to difficulty in culturing *leptospira*. Other methods for diagnosis are polymerase chain reaction and culturing the organism itself. Enzyme linked immunosorbent assay (ELISA) of IgM has a sensitivity of 89.9% and specificity of 97.4% [1].

Leptospirosis is usually a self-limiting disease but early use of antibiotics can shorten duration of illness, reduce disease severity and expedite recovery. Treatment should be initiated before serologic affirmation. The drug of choice is penicillin and cephalosporins while doxycycline is recommended for mild disease and for post-exposure prophylaxis. The use of penicillin may be complicated by Jarich-Herxheimer reaction. The reaction occurs when large quantities of endotoxins are released into the circulatory system as a result of bacterial death with antibiotic treatment. Typically release of endotoxins associated with the death of these bacteria occurs faster than the removal of the toxins by body [13]. This likely explains the decline in our patient’s clinical condition 24 h after initiating antibiotic therapy that led to the need for brief inotropic support.

In our case, a management dilemma was faced, as there was a clear indication for PEX but the potential of serious complications associated with PEX in probable underlying infection was also taken into consideration. There has been emerging evidence for PEX as a therapeutic measure in severe leptospirosis or when a Jarich-Herxheimer reaction develops [3, 13–15]. PEX can extract the excess of bacterial products, cytokines and inflammatory mediators [13]. The survival among those treated with plasma exchange was 77% compared with 17% in those treated with supportive treatment alone [14]. Our patient continued with PEX and antibiotics with significant improvement in the laboratory biomarkers and reflected by his overall clinical condition.

## Conclusion

The case highlights the importance of exploring other possible causes especially systemic infections in patients with suspected TTP/HUS as it would allow prompt treatment and possibly to avoid unnecessary therapeutic PEX treatment. A triad presentation of fever, jaundice and acute kidney injury should alert the physician of a probable diagnosis of leptospirosis especially in countries where this disease is perceived as low risk and can be easily overlooked. A disproportionate liver injury, which cannot be explained by haemolysis alone, should trigger one to re-evaluate the diagnosis and management course. Prompt recognition and administration of antibiotics is mandatory for reducing the risk of mortality.

## Abbreviations

AKI: Acute kidney injury
ALP: Alkaline Phosphatase
ALT: Alanine aminotransferase
BP: Blood pressure
ELISA: Enzyme-linked immunosorbent assay
HUS: Haemolytic uraemic syndrome
MAT: Microscopic agglutination test
PEX: Plasma exchange
TTP: Thrombotic thrombocytopenia
UK: United Kingdom
